# Inositol pyrophosphates mediated the apoptosis induced by hypoxic injury in bone marrow-derived mesenchymal stem cells by autophagy

**DOI:** 10.1186/s13287-019-1256-3

**Published:** 2019-06-03

**Authors:** Jingyu Deng, Chao Yang, Yong Wang, Ming Yang, Haixu Chen, Hongjuan Ning, Chengzhu Wang, Yanjun Liu, Zheng Zhang, Taohong Hu

**Affiliations:** 1 Postgraduate Training Base in Rocket Army Special Medical Center of the PLA, Jinzhou Medical University, Jinzhou, 121001 Liaoning China; 2 Department of Cardiology, The Rocket Army Special Medical Center of the PLA, Beijing, 100088 China; 3 Department of Blood Transfusion, The Rocket Army Special Medical Center of the PLA, Beijing, 100088 China; 4 Department of Nuclear Medicine, the Fifth Medical Center, Chinese PLA General Hospital (Former 307th hospital of the PLA), Beijing, 100071 China; 5 Department of Ophthalmology, Li Ka Shing Faculty of Medicine, The University of Hong Kong, Hong Kong, 000000 SAR China; 6 Institute of Geriatrics & National Clinical Research Center of Geriatrics Disease, Chinese PLA General Hospital, Beijing, 100853 China; 7 Jinzhou Medical University, Jinzhou, 121001 Liaoning China

**Keywords:** Hypoxia, Bone marrow mesenchymal stem cells (BM-MSCs), Inositol pyrophosphates (IP7), Autophagy, Apoptosis, Akt/mTOR signaling pathway

## Abstract

**Objective:**

To investigate the potential effect of IP7 on the autophagy and apoptosis of bone marrow mesenchymal stem cells (BM-MSCs) caused by hypoxia.

**Methods:**

BM-MSCs isolated from adult male C57BL/6 mice were exposed to normoxic condition and hypoxic stress for 6 h, 12 h, and 24 h, respectively. Then, flow cytometry detected the characteristics of BM-MSCs. Furthermore, N6-(p-nitrobenzyl) purine (TNP) was administrated to inhibit inositol pyrophosphates (IP7). TUNEL assay determined the apoptosis in BM-MSCs with hypoxia. Meanwhile, RFP-GFP-LC3 plasmid transfection and transmission microscope was used for measuring autophagy. In addition, Western blotting assay evaluated protein expressions.

**Results:**

Hypoxic injury increased the autophagy and apoptosis of BM-MSCs. At the same time, hypoxic injury enhanced the production of IP7. Moreover, hypoxia decreased the activation of Akt/mTOR signaling pathway. At last, TNP (inhibitor of IP7) repressed the increased autophagy and apoptosis of BM-MSCs under hypoxia.

**Conclusion:**

The present study indicated that hypoxia increased autophagy and apoptosis via IP7-mediated Akt/mTOR signaling pathway of BM-MSCs. It may provide a new potential therapy target for myocardial infarction (MI).

## Introduction

Although the treatment of coronary heart disease has made rapid progress, ischemic heart disease induced by coronary artery occlusion is a key cause of morbidity and mortality around the world [1–4]. At present, treatment with stem cell has a potential effect on cardiovascular regeneration of ischemic heart disease. And it has been considered as a promising therapeutic method. Bone marrow mesenchymal stem cells (BM-MSCs) are widely applied for regenerative medicine because of their plasticity and effectiveness [5–8]. However, in practice, the poor activity and function of donor cells greatly limit the efficiency of stem cell transplantation [9]. Thus, excessive apoptosis, caused by harsh microenvironment, has been regarded as the main reason for the donor cell death [8].

Autophagy, a self-degrading process that responds to stress, plays a key effect on balancing sources of energy and resetting misfolded proteins. Therefore, autophagy is considered as a protective and adaptive mechanism to promote cell survival under normal conditions [10, 11]. However, most autophagosomes (over-autophagosome formation) are associated with autophagic cell death [12]. In the heart, a certain degree of autophagy is observed at baseline, and its expression is upregulated by pathological stimuli such as myocardial ischemia [13, 14]. Currently, our previous study demonstrated that hypoxia increased the autophagy and apoptosis of BM-MSCs time-dependently. Furthermore, autophagy regulated the increased apoptosis in BM-MSCs under hypoxia via AMPK/mTOR signal pathway [8].

Inositol polyphosphates are a key signaling molecules in cells [15]. Although most of the more than 30 inositol polyphosphates in mammalian cells have unknown physiological functions, a group of higher inositol phosphates including energetic pyrophosphate bonds and inositol pyrophosphates (IP7) has been identified [16–18]. IP7, generated by inositol hexakisphosphate (IP6) through Inositol hexakisphosphate kinases (IP6Ks), is related to diverse functions containing vesicle transport and chemotaxis [19–24]. Recently, Chakraborty et al. [25] found that IP6k1 gene knockout can increase Akt activity, confirming that IP7 is a physiological inhibitor of Akt signal pathway. In addition, study has also showed that hypoxic injury increased IP7 formation in MSCs, which inhibits Akt activation [26].

Previous research has found an increase in the inositol pyrophosphate signaling induces the increased autophagosomes. Furthermore, they thought that IP7 plays a key role in regulating autophagy [15]. However, the specific mechanism of IP7 and autophagy that contributes to apoptosis in MSCs under hypoxic injury is still unclear. Thus, our present study aimed to elucidate the potential effect of IP7 on the autophagy with hypoxic injury, which may provide optimal approaches to improve the therapeutic effect of MSCs for ischemic heart disease.

## Methods

### Animals

We selected adult male C57BL/6 mice as experimental animals (from the Laboratory Animal Research Center of Rocket Army Special Medical Center of Chinese People’s Liberation Army) to isolate BM-MSCs. Before any experiment, mice were placed into temperature-controlled animal facilities for 12 h of light/dark cycle (light cycle, 8:00 a.m. to 8:00 p.m.), with tap water and rodent chow provided 2 weeks ad libitum. The Animal Care and Use Committee of the Rocket Army Special Medical Center of the PLA (ID: 5034) approved all the experimental procedures. And experimental procedures were conformed to the Guidelines for the Nursing and Use of Experimental Animals published by the Press of the National Academy of Sciences.

### Isolation and culture of BM-MSCs

We used a modified procedure to isolate and expand of BM-MSCs [27]. In brief, we flushed bone marrow from the femoral and tibia with phosphate-buffered saline (PBS). Dulbecco’s modified Eagle’s medium (DMEM) supplemented with 20% fetal bovine serum (FBS) and 1% penicillin/streptomycin medium re-suspended cell granules after being passed through a 70-mm filter and centrifugation at 1200 rpm for 5 min. Different treatments with third-passage MSCs avoided contamination with other types of cell.

### Characteristics of BM-MSCs

Flow cytometry tested characteristics of BM-MSCs, such as CD34-, CD45-, CD44+, and CD90+. And we performed in vitro differentiation assay [26, 27]. Briefly, a FACS Calibur system (BD) treated BM-MSCs according to the manufacturer’s guideline after being incubated with monoclonal PE-binding antibodies against special CD markers (BD, San Jose, CA, USA) for 1 h.

The in vitro differentiation assays were used for performing MSCs [26, 27]. Adipogenic media (aMEM containing 10% FCS, 50 mM indomethacin, 1% antibiotics, 1 mM dexamethasone, and 0.5 mM IBMX) were added into the confertus layer of MSCs for 21 days in order to perform adipogenic differentiation. Then, a working solution of Oil Red O stained the adipogenic cells for 15 min at room temperature. Twenty-one-day osteogenic differentiation of MSCs was induced concurrently by osteogenic medium (OM, 10% FBS, 10 mM b-glycerophosphate, 1000 nM dexamethasone, and 0.2 mM ascorbic acid in a-MEM). Alizarin red S staining was used for estimating the degree of extracellular matrix calcification. Furthermore, we induced chondrogenic differentiation of BM-MSCs as described previously [28]. In brief, chondrogenic medium (containing 50 mg/l ascorbic acid, 100 nmol/l dexamethasone, 1% fetal bovine serum, low-glucose DMEM, 100 mg/l sodium pyruvate, 40 mg/l l-proline, and 1.0% indometacin) was added into BM-MSCs for 21 days. Meanwhile, Alcian blue stain (0.5 ml, 30 min at room temperature) was performed to evaluate the chondrogenic differentiation.

### Hypoxia/serum deprivation injury

Hypoxia/ serum deprivation injury was used to perform the hypoxic stress of BM-MSCs as described previously [26]. In short, BM-MSCs were cultivated with 20% FBS. After being replaced in Hanks buffer, BM-MSCs were treated with hypoxic condition (94% N_2_–5% CO_2_–1% O_2_) with an anaerobic system (Thermo Forma) at 37 °C for 6, 12, and 24 h, respectively. BM-MSCs in the control group were maintained at normal (95% air–5% CO2) condition for equal periods.

### Treatment of cells

To regulate the autophagy level in BM-MSCs, IP7 inhibitor N6-(p-nitrobenzyl) purine (10 μM, TNP) was added to further explore the molecular mechanism of autophagy in BM-MSCs with normal and hypoxic treatments. Treatments of cells were done in duplicate [27].

### Determination of apoptosis in BM-MSCs

Terminal-deoxynucleotidyl transferase mediated-dUTP nick-end labeling (TUNEL) assay confirmed the apoptosis of MSCs with an assay kit (In Situ Cell Death Detection Kit; Roche Diagnostics) according to the manufacturer’s guideline [29]. In brief, BM-MSCs were incubated with TdT and fluorescein-labeled dUTP for 45 min at 37 °C after different treatments. Then, 4,6-diamidino-2-phenylindole (DAPI) was used for identifying nucleus. We took photographs with confocal microscopy (Olympus Fluoview 2000). At the same time, we calculated the percentage of apoptotic cells. And then, we counted five random fields for analysis in each group. We did all assays blindly.

### Measurement of autophagy in BM-MSCs

For assessing the autophagy of MSCs, we transfected the mRFP-GFP-LC3 plasmids (Hanbio biotechnology Co., Ltd., Shanghai, China) into BM-MSCs as described previously [30, 31]. In brief, we took microphotographs of BM-MSCs after different treatments. Furthermore, we counted five random fields and calculated the percentages of cells with RFP-GFP-LC3 punctate. We did all assays blindly. At the same time, Western blotting detected the expressions of LC-3 and autophagy-associated protein (P62 and Beclin-1).

### Western blot assay

We harvested and dissolved BM-MSCs in protein lysis buffer (Sigma). Equivalent protein (50 mg/lane) was separated by electrophoresis on 12% SDS-PAGE gels at 120 V for 90 min and then electrophoretically transferred onto PVDF membranes at 100 mV for 1.5 h. Cellular membranes were subjected to immune-blotting with primary antibodies overnight at the temperature 4 °C after blocked in 5% nonfat dry milk (BD Biosciences) at room temperature for 1 h. After incubation with appropriate secondary antibody binding to horseradish peroxidase, we used an enhanced chemiluminescene system (Amersham Bioscience) to visualize blots bands. Furthermore, we used VisionWorks LS, version 6.7.1, to determine densitometric analysis of Western blots [8].

The primary antibodies were used: rabbit anti-mouse LC-3 (1:500, Cellular Signal Technology), rabbit anti-mouse Beclin-1 (1:500, Cellular Signaling Technology), rabbit anti-mouse P62 (1:500, Cellular Signal Technology), rabbit anti-mouse phosphorylated (Thr172) and rabbit anti-mouse total AKt (1:200, Abcam), rabbit anti-mice mTOR (1:500, Abcam), rabbit anti-mouse p-mTOR (1:200, Abcam), rabbit anti-mouse p70S6k (1:500, Abcam), rabbit anti-mouse S6 (1:500, Abcam), rabbit anti-mice p-p70S6k (1:200, Abcam), rabbit anti-mouse p-S6 (1:200, Abcam), rabbit anti-mouse caspase-3 and cleaved caspase-3 (1:500, Abcam), and rabbit anti-mouse β-actin (12,000, Abcam).

### Statistics analysis

Our results were shown with the mean ± SEM. Prism 5.0 (GraphPad Software Inc., San Diego, CA, USA) was used for the statistical analyses. The different groups of this study were compared by the homogeneity tests and one-way ANOVA. *P* value < 0.05 was considered as a statistical significance.

## Results

### Biologic character of BM-MSCs

**Fig. 1:**
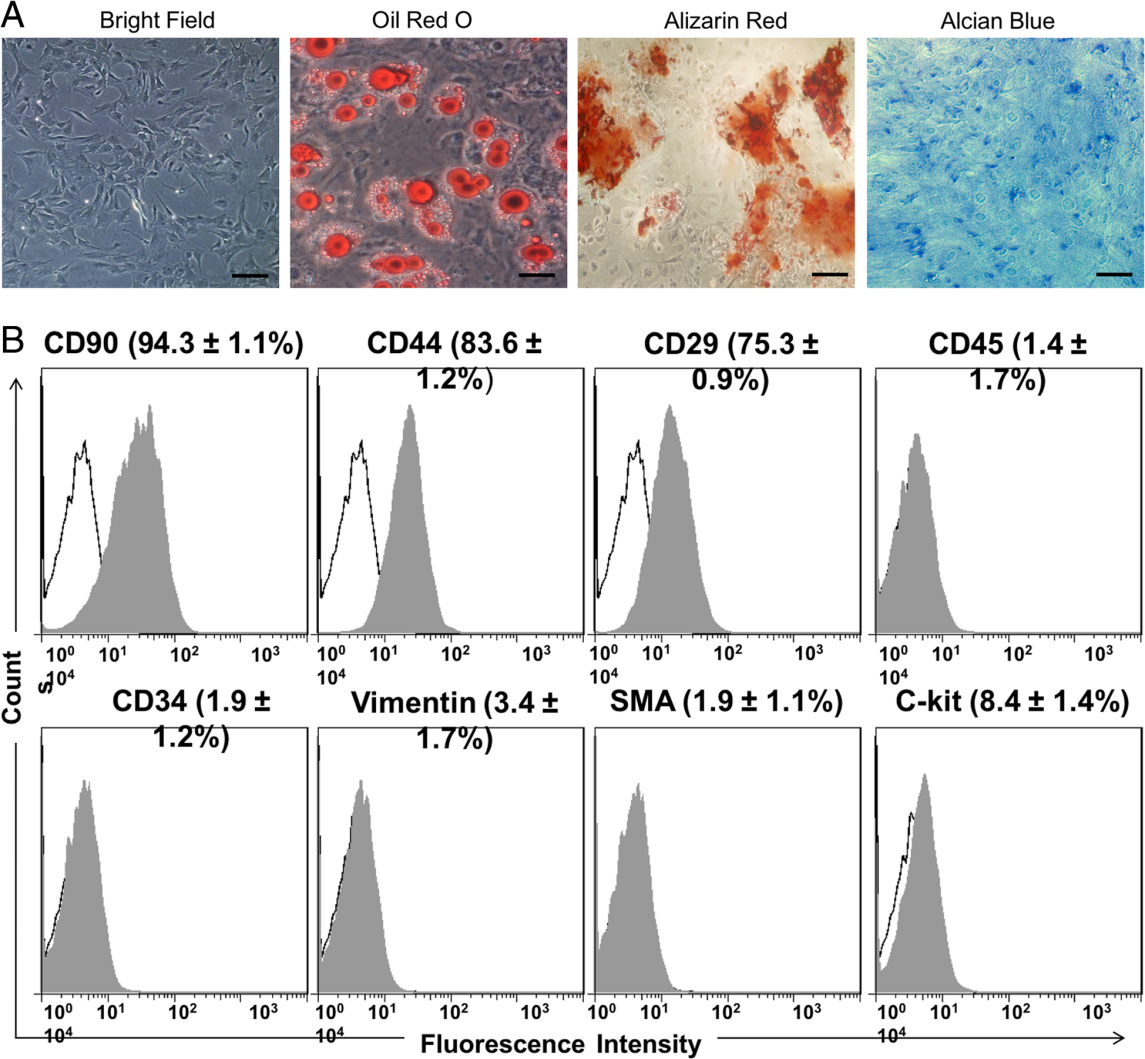
Character of BM-MSCs. **a** BM-MSCs had fibroblast-like shapes. Oil Red O staining, Alizarin Red S staining, and Alcian blue staining detected Adipogenesis, osteogenesis, and chondrogenesis of BM-MSCs respectively (scale bar, 100 μm). **b** Results of flow cytometry demonstrate that BM-MSCs were negative for CD45, CD34, Vimentin, SMA, and c-kit and positive for CD90, CD29, CD44

We used in vitro multi-lineage differentiation and flow cytometry analysis to identify and analyze the character of BM-MSCs. We observed BM-MSCs of fibroblast-like shapes (Fig. 1a). To set up the multi-lineage differentiation proficiency of BM-MSCs, we used adipogenic and osteoblastogenic media to incubate cells for 21 days respectively. The result of Oil Red O staining revealed that about 70% of BM-MSCs possessed an adipocytes phenotype. Moreover, we observed BM-MSCs differentiating into osteogenic cells from the images of alizarin red S staining for calcium deposit. Furthermore, Alcian blue staining indicated the chondrogenic differentiation of BM-MSCs (Fig. 1a). All of these results demonstrated that BM-MSC had proficiency of multi-lineage differentiation. Results of flow cytometry analysis revealed that MSCs were homogeneously positive for BM-MSC markers CD29, CD44, and CD90 and negative for Vimention, c-Kit, SMA, CD45, and CD34 (Fig. 1b).

### Hypoxia increased autophagy of MSCs

**Fig. 2:**
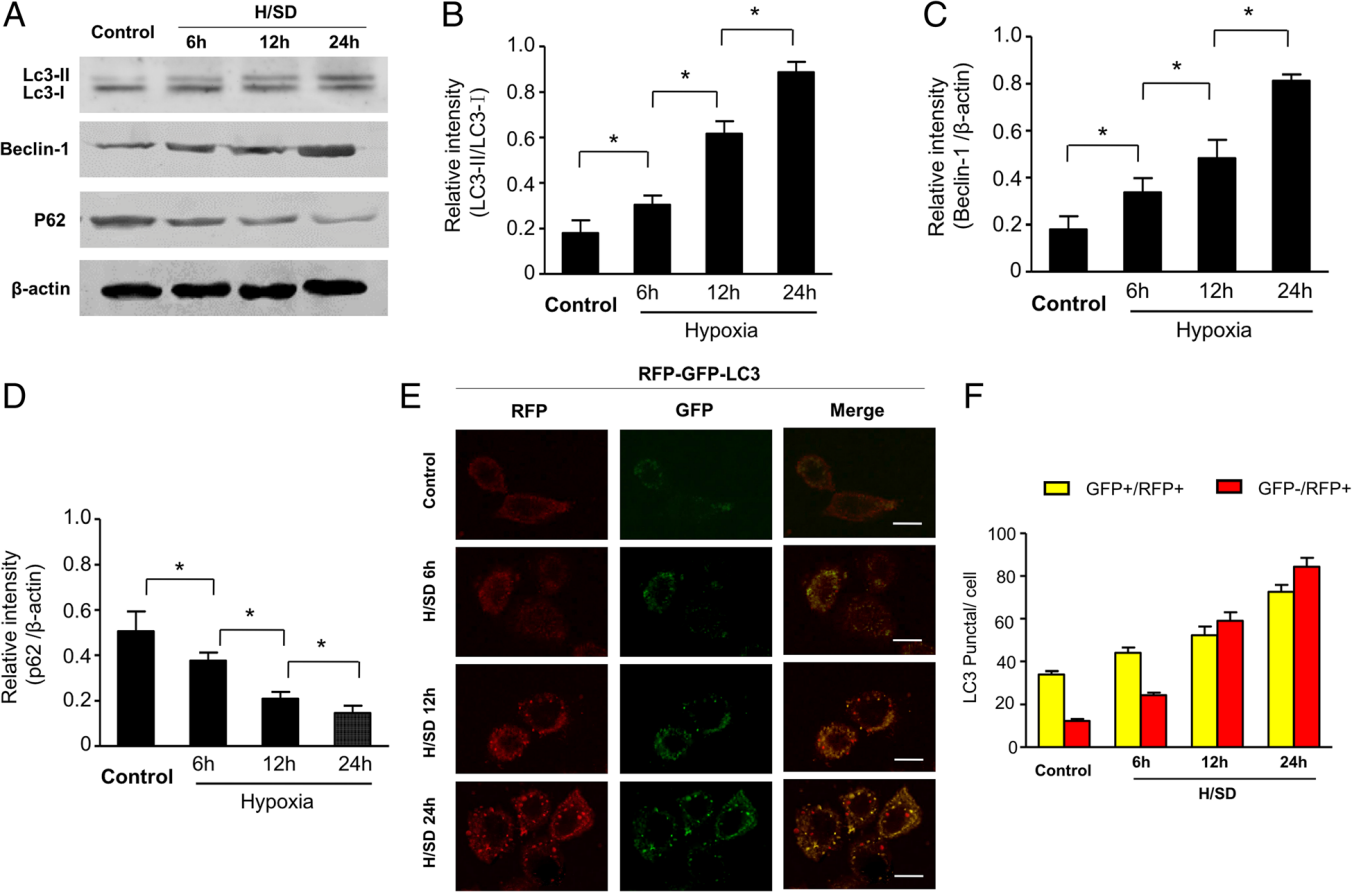
Hypoxic stress increased autophagy in BM-MSCs. **a** Representative Western blots of LC3-I/LC3-II, Beclin-1, and P62 in BM-MSCs subjected to normal and hypoxic conditions. Quantification of the protein expression levels of LC3-II/ LC3-I (**b**), Beclin-1 (**c**), and p62 (**d**) at the indicated time points. **e** Representative immunofluorescence images of red fluorescent protein (RFP)-green fluorescent protein (GFP)-LC3 (red fluorescence) in BM-MSCs under normal conditions and H/SD (scale bars, 10 mm). **f** Quantification of autophagy was presented as the numbers of puncta in LC-3 positive cells (*n* = 5, *p* < 0.05). Data are expressed as the means ± SEM; *n* = 5; *p < 0.05

We assessed the expressions of autophagy regulating signal proteins to explore the autophagy level under hypoxic condition. Western blot revealed that the expression of LC3-II and Beclin-1 was increased significantly in BM-MSCs with hypoxia time-dependently, while the expressions of p62 were decreased (Fig. 2a–d). Moreover, we transfected BM-MSCs with RFP-GFP-LC3 and tracked the expressions of LC3. After hypoxic treatment, not only were the numbers of green and red puncta both significantly higher (Fig. 2e), but the yellow dots were also typically increased in the merged images, which show that autophagosomes were increased. These results imply that autophagic flux of BM-MSCs was enhanced in hypoxic condition. Meanwhile, semi-quantitative analysis revealed that the numbers of puncta in LC-3-positive cells gradually increased along with the exposure time to hypoxic treatment compared with the control group (Fig. 2f). Taken together, results suggest that hypoxia increased the autophagy in BM-MSCs.

### Hypoxic stress induced apoptosis in MSCs

**Fig. 3:**
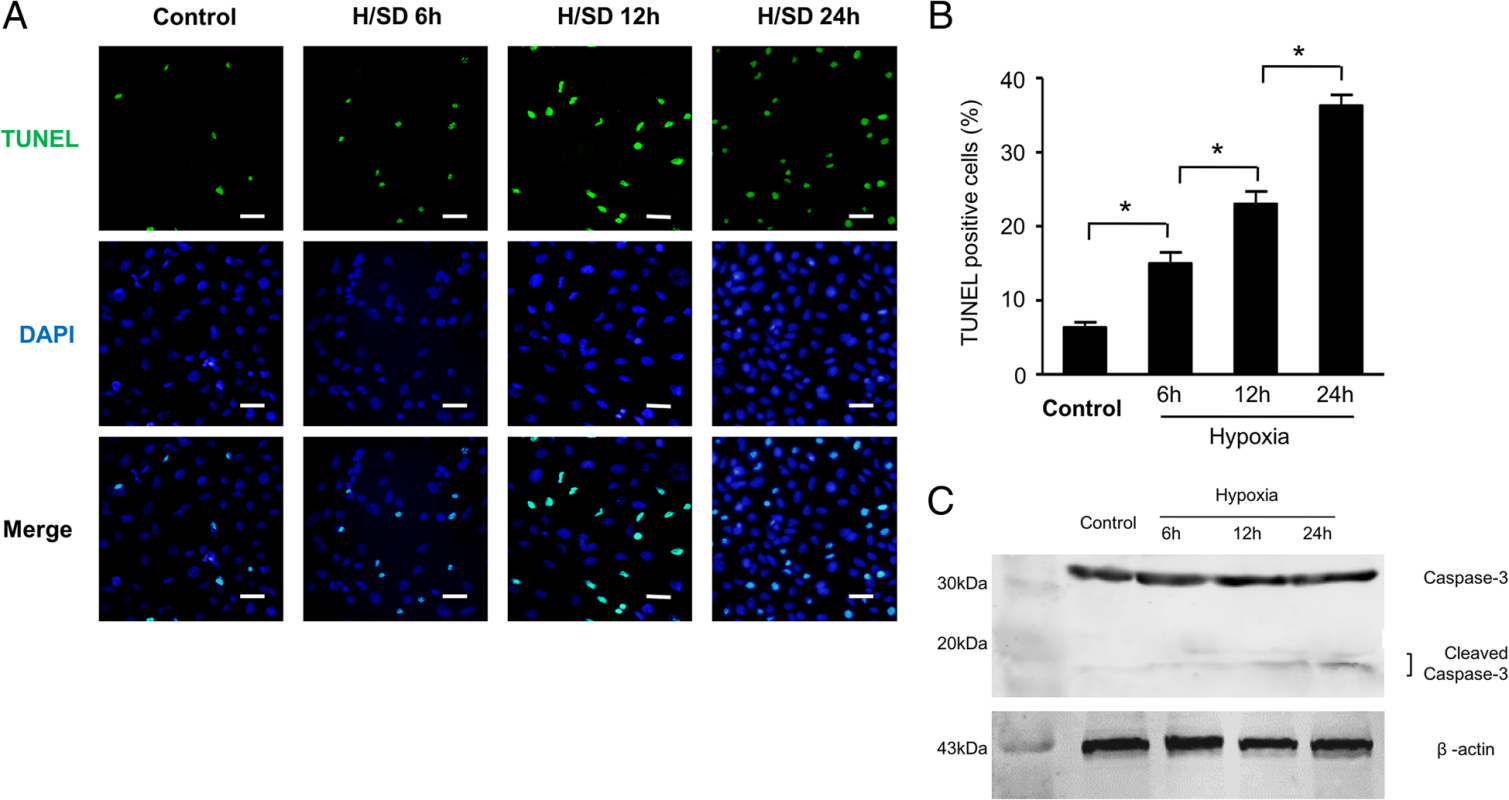
Hypoxia induced apoptosis of BM-MSCs. **a** Representative TUNEL images of BM-MSCs under normal conditions and H/SD (scale bars, 20 μm). **b** Quantification of the apoptotic BM-MSCs was presented as the percentage of apoptotic cells (*n* = 5, *p < 0.05). **c** Representative Western blots of cleaved caspase-3

We evaluated the apoptosis of MSCs to detect the effects of hypoxic conditions on apoptosis by TUNEL assay. The representative immunofluorescence images (Fig. 3a) revealed that compared with BM-MSCs with normoxia, positive cells in TUNEL assay were significantly increased under hypoxia for 6 h, 12 h, and 24 h, respectively. Moreover, we observed that the percentages of TUNEL-positive BM-MSCs under hypoxic condition for 6 h, 12 h, and 24 h were 15.0 ± 2.6%, 23.0 ± 3.1%, and 36.3 ± 2.5% respectively, significantly higher than that in normoxic group (6.3 ± 1.3%, *p* < 0.05, Fig. 3b) from quantitative analysis. In addition, we saw the increased expression of cleaved caspase-3 by H/SD time-dependently (Fig. 3c). Taken together, our data suggest that hypoxic stress induces apoptosis in BM-MSCs time dependently.

### Hypoxic treatment increased IP7 production in MSCs

**Fig. 4:**
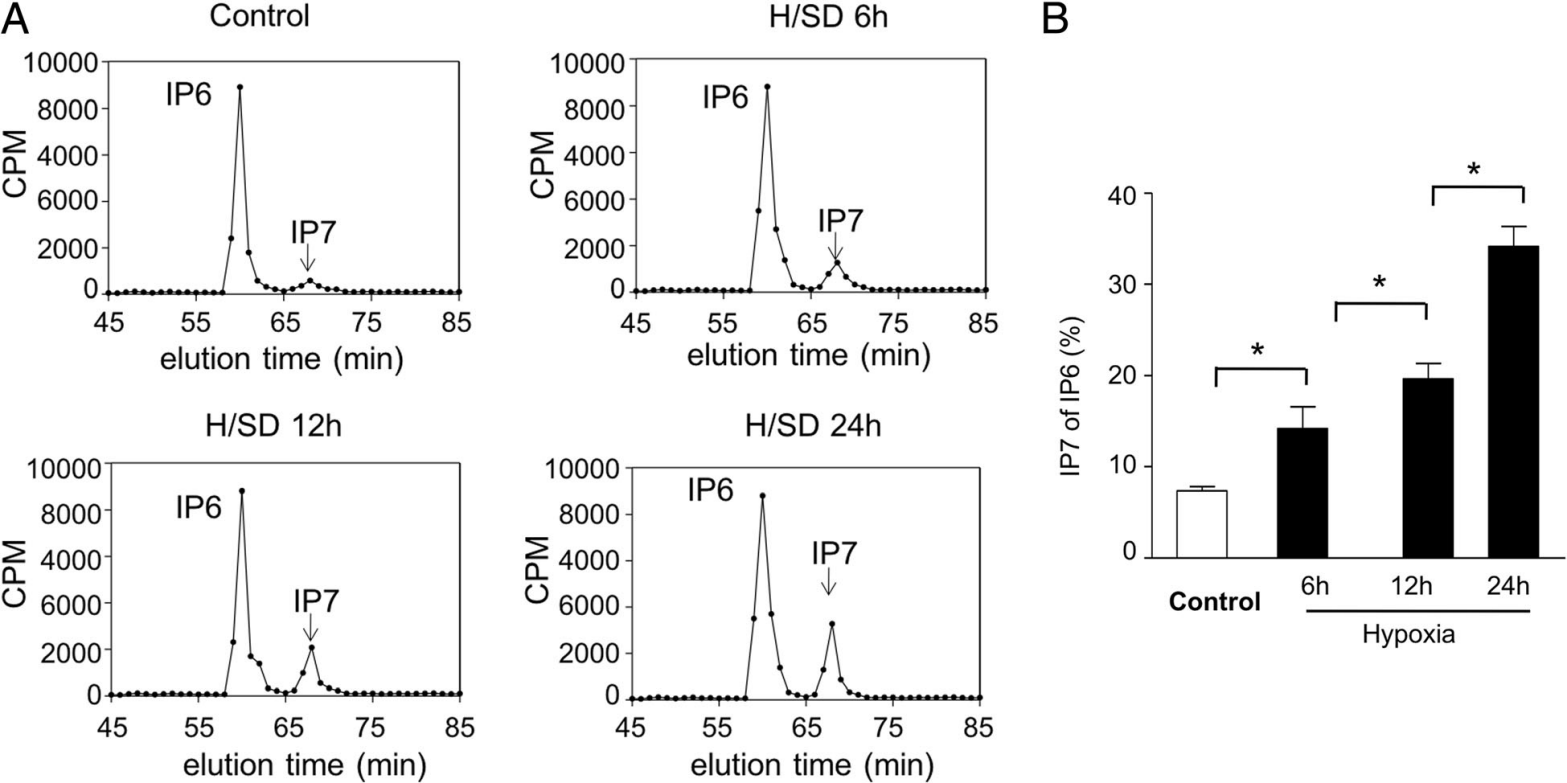
Hypoxic injury increased IP7 production in BM-MSCs. **a** HPLC profiles of inositol phosphates isolated from MSCs under normal and hypoxic conditions. **b** Quantitative analysis of IP7 production as a percentage of IP6 in MSCs (*n* = 5, *p < 0.05)

To detect the activity of IP6Ks and synthesis of IP7, the content of inositol polyphosphate was determined by HPLC. Compared with normoxic condition, IP7 production was significantly increased in BM-MSCs with hypoxic conditions for 6 h, 12 h, and 24 h, respectively (Fig. 4a). Quantitative analysis demonstrated that the percentage of IP7 in IP6 was significantly increased in BM-MSCs under hypoxia for 6 h, 12 h, and 24 h, respectively (14.20 ± 4.12% in hypoxia 6 h group, 19.66 ± 2.95% in hypoxia 12 h group, 34.17 ± 3.75% in hypoxia 24 h group vs. 7.34 ± 0.81% in normal condition *P* < 0.05) (Fig. 4b). In summary, these results demonstrate that hypoxia increases the production of IP7 in BM-MSCs.

### Hypoxia decreased the activity of the Akt/mTOR signal in BM-MSCs

**Fig. 5:**
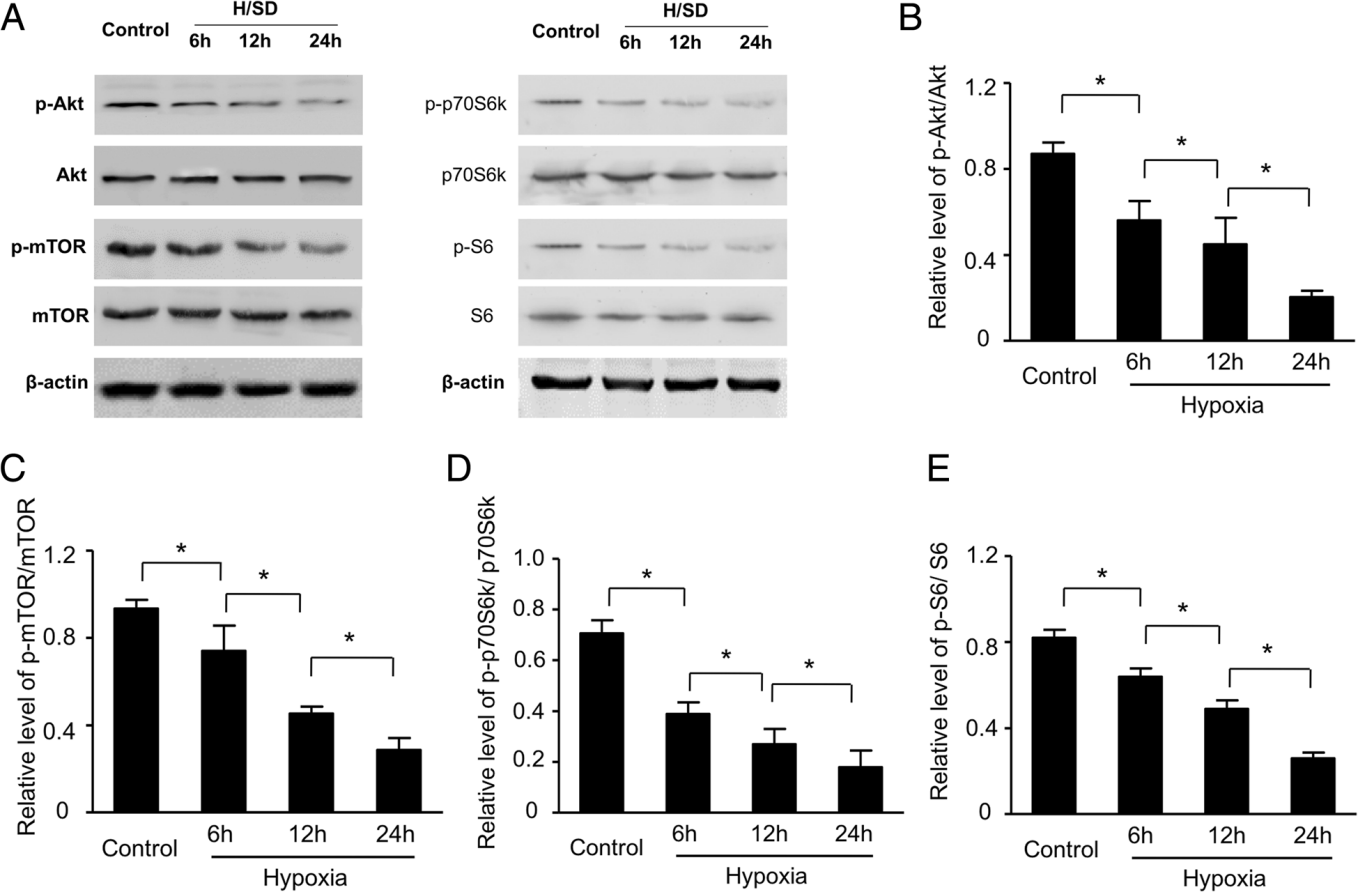
Hypoxic stress reduced the activity of the Akt/mTOR signaling pathway. **a** Representative Western blots of p-Akt/Ak, p-mTOR/mTOR, p-p70S6 K/p70S6 K, p-S6/S6, and β-actin in BM-MSCs subjected to normal condition and hypoxic injury for 6, 12, and 24 h, respectively. Quantification of the protein expression levels of p-Akt (**b**), p-mTOR (**c**), p-p70S6 K/p70S6 K (**d**), and p-S6/S6 (**e**) (*n* = 5, *p < 0.05)

To investigate the effect of hypoxic stress on the activation of the Akt and mTOR signaling pathway in hypoxic condition, we examined the expressions of Akt and mTOR by Western blot assay. As the typical Western blot results and semi-quantitative analyses shown in Fig. 5a, b, compared with normoxic condition, the expression of phospho-Akt (Ser473) of BM-MSCs were dramatically decreased under hypoxic conditions for 6, 12, and 24 h, respectively. Meanwhile, the expression of phospho-mTOR (Ser2448) reduced in BM-MSCs with hypoxic treatments (Fig. 5a, c). In addition, hypoxic injury also remarkably decreased the downstream effectors of mTOR signal pathway, such as the ribosomal S6 protein (S6) and phosphorylation of p70 ribosomal S6 subunit kinase (p70S6K) (Fig. 5a, d, and e). In summary, all data show that hypoxia plays a vital role in decreasing the activity of the Akt/mTOR signal pathway of BM-MSCs.

### TNP restrained IP7 production in BM-MSCs

**Fig. 6:**
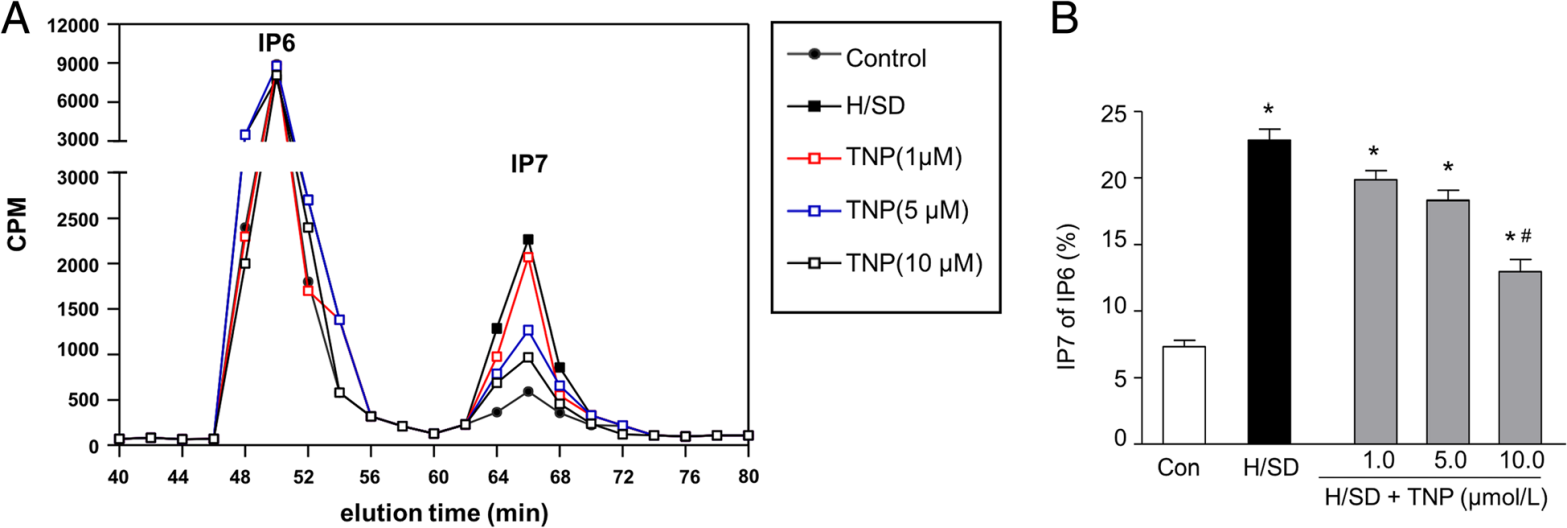
Activity of IP6Ks and synthesis of IP7 after hypoxia and serum deprivation (H/SD) injury. **a** High-performance liquid chromatography (HPLC) profiles of inositol phosphates isolated from MSCs labeled with [2-3H] inositol and stimulated with TNP (0.5, 1, 5, and 10 μmol/L) at 37 °C for 2 h. **b** Quantitative analysis of a histogram indicates percentage of IP7 in IP6 of MSCs. Data were expressed by mean ± SEM. *n* = 5, *p < 0.05 vs. control (normoxia), ^#^*p* < 0.05 vs. H/SD group. CPM counts per minute

To detect the effect of IP7 on the hypoxic injury of MSCs, a pharmacological IP6Ks inhibitor TNP was administrated to decrease IP7 synthesis. The HPLC results revealed that hypoxia and serum deprivation (H/SD) injury increased production of IP7 compared with the control group (Fig. 6a). However, different doses of TNP inhibited IP7 production. Meanwhile, the formation of IP7 was dramatically repressed by the presence of TNP at 10 μmol/L. Additionally, quantitative analysis of a histogram in Fig. 6b showed that H/SD injury accumulated the percentage of IP7 in IP6 (*P* < 0.05), compared with the control group. Moreover, the percentage of IP7of IP6 was dramatically inhibited by TNP at 10 μmol/L (***P*** < 0.05).

### TNP decreased apoptosis in BM-MSCs

**Fig. 7:**
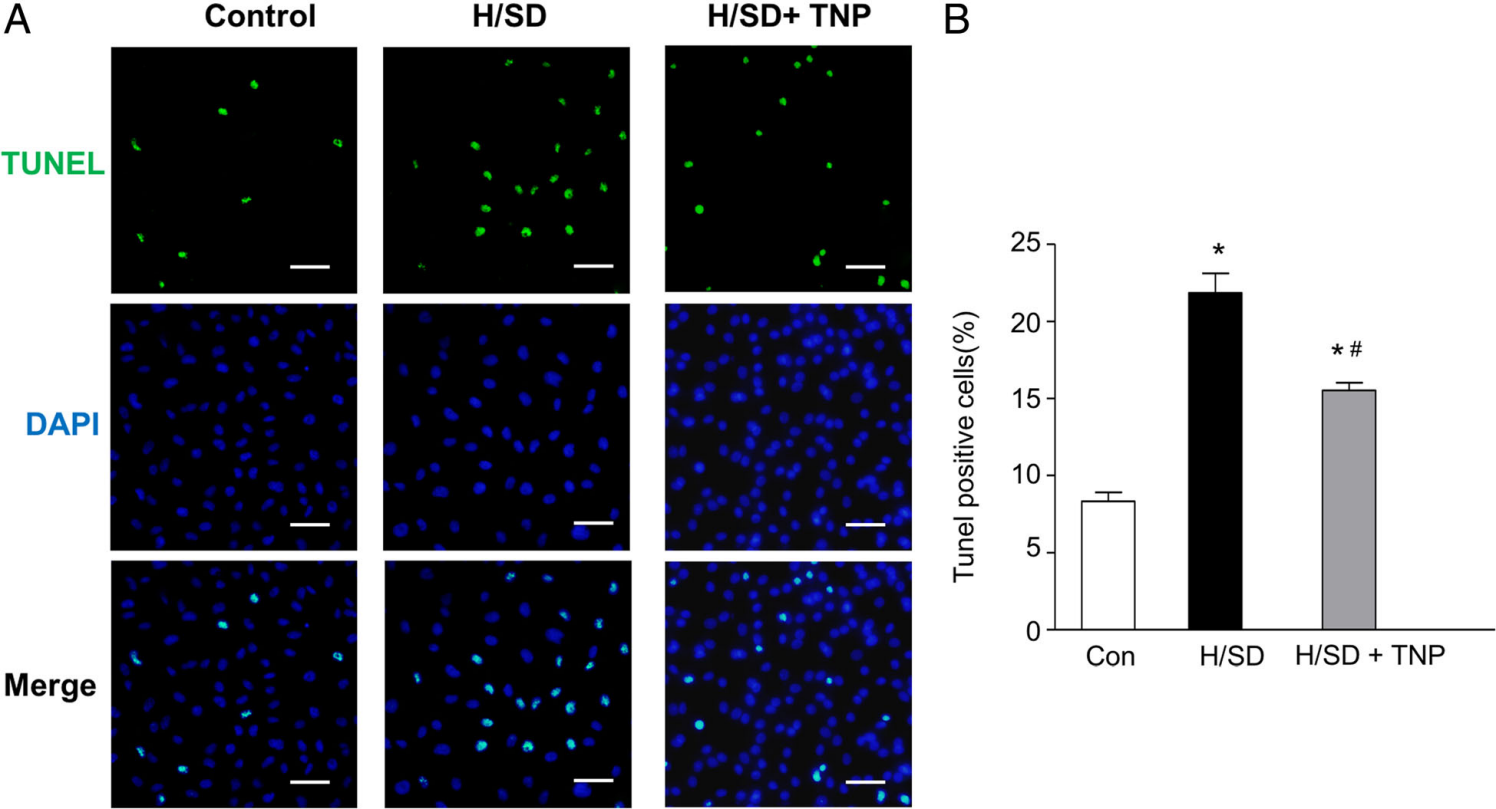
TNP treatment reduced apoptosis of BM-MSCs. **a** Representative TUNEL images of BM-MSCs with normoxia, H/SD, and TNP treatment (scale bars, 20 μm). **b** Quantification of the BM-MSCs of apoptosis was the percentage of apoptotic cells. Data were expressed with mean ± SEM. *n* = 5, *p < 0.05 vs. control (normoxia), ^#^*p* < 0.05 vs. H/SD group

To discover the effects of IP7 on the apoptosis induced by hypoxia, we evaluated the apoptosis of BM-MSCs after TNP administration by the TUNEL assay. The representative immunofluorescence images of TUNEL (Fig. 7a) demonstrated that compared with BM-MSCs in control, TUNEL-positive cells were significantly increased under hypoxia. Moreover, TUNEL-positive cells were reduced after TNP treatment compared with that in H/SD group. In addition, the quantitative analysis indicated that H/SD significantly induced apoptosis of MSCs (21.87 ± 2.21% vs. 8.37 ± 1.02% in normal condition, *p* < 0.01). However, TNP at 10 μM significantly decreased the percentage of apoptotic MSCs compared with H/SD group (15.53 ± 0.84% vs. 21.87 ± 2.21%, *p* < 0.05) (Fig. 7b).

### TNP pre-treated reduced BM-MSC autophagy

**Fig. 8:**
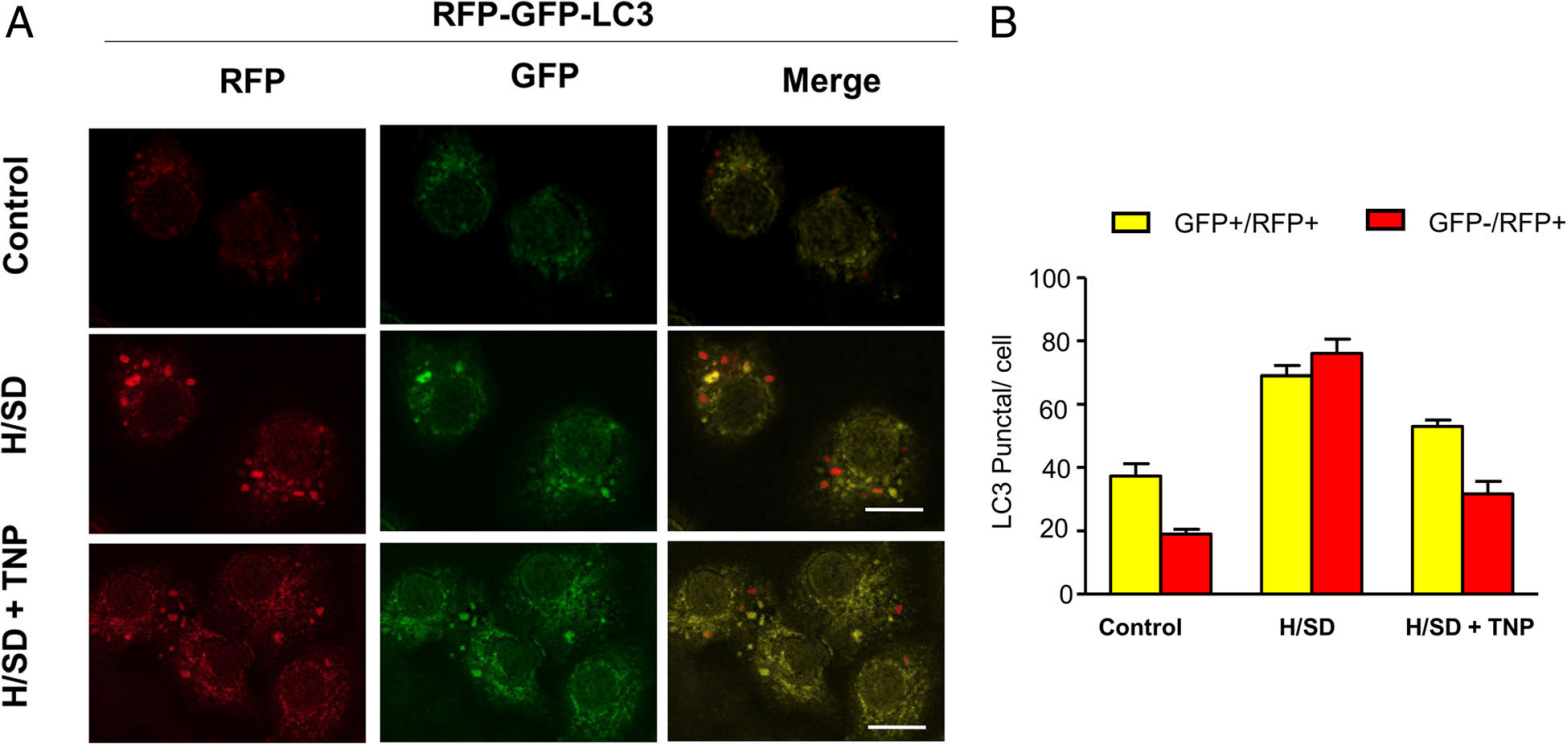
TNP pretreatment reduced autophagy in BM-MSCs. **a** Representative immunofluorescence images of red fluorescent protein (RFP)-green fluorescent protein (GFP)-LC3 (red fluorescence) in BM-MSCs under normal conditions, H/S, and TNP treatment (scale bars, 10 mm). **b** Quantification of autophagy was presented as the numbers of puncta in LC-3-positive cells (*n* = 5, *p* < 0.05). Data are expressed by the means ± SEM; *n* = 5; *p* < 0.05

To investigate the effects of IP7 on the apoptosis induced by hypoxia, we evaluated the autophagy of BM-MSCs after TNP administration. Immunofluorescence images in Fig. 8a showed that the numbers of green and red dots both reduced after TNP pre-treatment and the yellow puncta were also typically decreased after TNP group vs. H/SD group in the merged images. Furthermore, the result of quantitative analysis revealed that compared with that in H/SD group, the numbers of puncta in LC-3-positive cells decreased in TNP treatment (Fig. 8b). Overall, our results demonstrated that TNP restrained autophagy in BM-MSCs.

### TNP treatment increased the activity of the Akt/mTOR signal in BM-MSCs

**Fig. 9:**
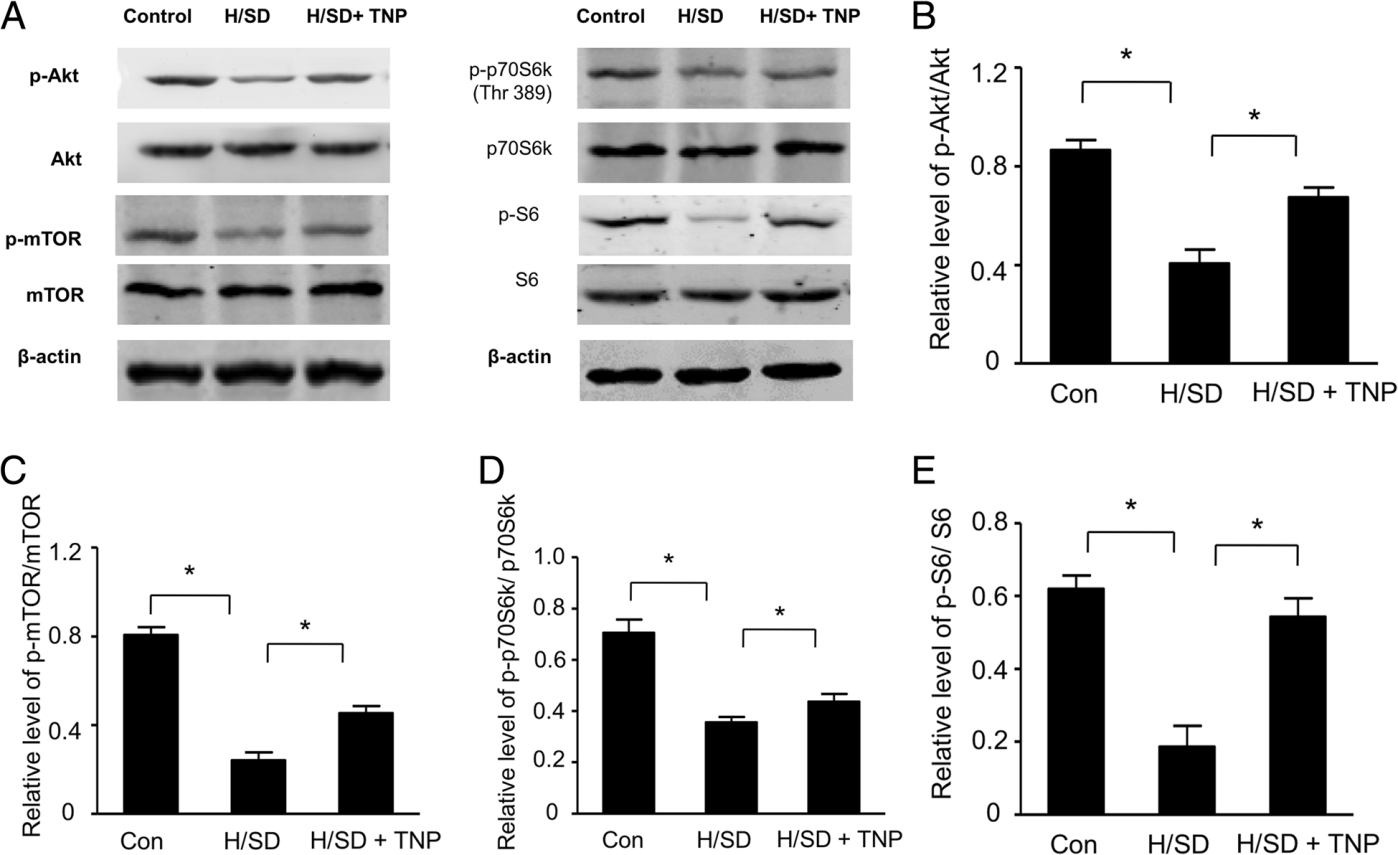
TNP enhanced the activity of the Akt/mTOR signaling pathway. **a** Western blots of p-Akt/Ak, p-mTOR/mTOR, p-p70S6 K/p70S6 K, p-S6/S6, and β-actin of BM-MSCs subjected to normal condition, H/SD injury, and TNP treatment. Quantification of the protein expression levels of p-Akt (**b**), p-mTOR (**c**), p-p70S6 K/p70S6 K (**d**), and p-S6/S6 (**e**) (*n* = 5, *p < 0.05)

We detected the effect of TNP pre-treatment on the activity of the Akt and mTOR signal with Western blot assays. The results of Western blot and semi-quantitative analyses (Fig. 9a, b) showed that the expression levels of phospho-Akt (Ser473) were significantly decreased under H/SD condition. However, the expression levels of phospho-Akt (Ser473) were remarkably increased in BM-MSCs with TNP treatment compared to that in H/SD BM-MSCs. Meanwhile, the expression of phospho-mTOR (Ser2448) reduced in BM-MSCs with hypoxia. However, we observed the increased expression of phospho-mTOR (Ser2448) in BM-MSCs with TNP treatments (Fig. 9a, c). In addition, hypoxic injury also significantly depressed the phosphorylations of the downstream effectors of mTOR signal, such as ribosomal S6 and p70S6K. Nevertheless, TNP pre-treated increased the expressions of p70S6K and S6 of BM-MSCs (Fig. 9a, d, and e). Taken together, these data indicated that TNP improved the activation of the Akt/mTOR signal in BM-MSCs.

## Discussion

**Fig. 10:**
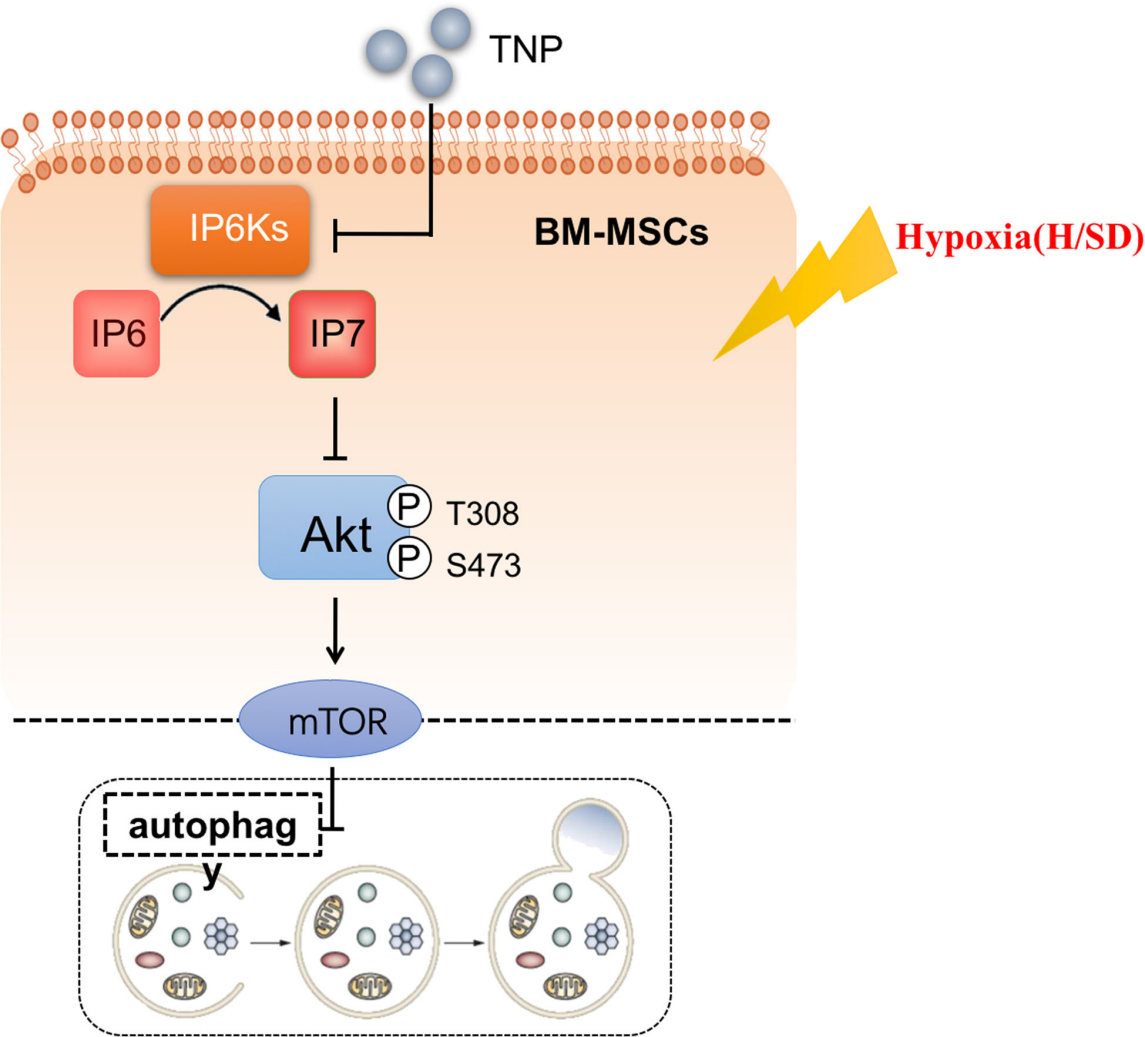
Proposed mechanism of autophagy and apoptosis of BM-MSCs caused by hypoxic stress. Hypoxia increases the production of IP7, which inhibits activation of Akt and subsequently restrains mTOR. Moreover, repressed mTOR directly enhances autophagy. Therefore, hypoxic injury induces autophagy regulating apoptosis via Akt/mTOR signaling pathway mediated by IP7 in BM-MSCs

Although bone marrow mesenchymal stem cells (BM-MSCs) were considered as a potential cellular source for therapy of myocardial infarction (MI) [32], the poor survival rate of transplanted cells was a major challenge for therapeutic efficacy [33]. Previous studies have found that autophagy may be a mechanism to regulate the death of MSCs after transplantation. Thus, the key to improve the treatment of MSCs is to regulate autophagy and promote the survival of MSCs after transplantation. IP7 is a newly discovered upstream signaling molecule regulating Akt, which play a crucial role in regulating autophagy. In our present study, we observed that hypoxia increased the autophagy and apoptosis of BM-MSCs time-dependently. Moreover, hypoxic stress also increased IP7 production in MSCs. Furthermore, restraining IP7 production by TNP reduced the autophagy and apoptosis of BM-MSCs caused by hypoxia. In short, our results for the first time implied that selective inhibition of IP6Ks has the protective effects of MSCs from hypoxic injury presumably via activity of the Akt signal. Furthermore, IP6Ks inhibition may be a potential strategy of optimizing mesenchymal stem cells therapy for patients with MI (Fig. 10).

Though autophagy plays an essential role in maintaining homeostasis or normal function in basic catabolic mechanism, it also can be dramatically induced and upregulated by unfavorable stimulus, such as hypoxia [34, 35]. Therefore, autophagy, a paradox that protects and damages cell survival, depends on the environment. Our previous studies demonstrated that autophagy was increased in BM-MSCs under hypoxic injury [8, 36]. In our study, we transfected BM-MSCs with RFP-GFP-LC3 to evaluate the autophagy of MSCs. The red LC3B puncta indicates a successful fusion among autophagosomes and lysosomes, because the acidic pH of the lysosome inhibits the acid-sensitive GFP (green color) on LC3B. Moreover, the yellow LC3B points show an impaired fusion between autophagosome and lysosome, because the acid-sensitive GFP will remain intact together with the acid-insensitive RFP (red color) [36]. Our results revealed that hypoxic stress increased the punctate LC3 in BM-MSCs, which enhances the expression of LC3-I/LC3-II and Beclin-1. Moreover, previous studies demonstrated that the p62 protein becomes incorporated into the completed autophagosome and is degraded in autolysosomes. Therefore, the expression of p62 is negatively correlated with autophagic activity [37, 38]. Our result showed that the expression of p62 was decreased under hypoxia. Taken together, these results indicated that hypoxia increased the autophagy of MSCs. Furthermore, the TUNEL assay showed that apoptosis in BM-MSCs was significantly increased under hypoxia time-dependently. Meanwhile, we also observed the increased expression of cleaved caspase-3 by H/SD time-dependently. In summary, these data suggested that hypoxia increased autophagy and apoptosis of BM-MSCs, which shows no difference with previous study [8, 39].

Akt signal pathway played an important role in regulating energy metabolism, cell survival, and oxidative stress [40]. Studies showed that mTOR repressed the cellular catabolic pathway, containing autophagy [41]. Similarly, our results demonstrated that hypoxia decreased significantly the expressions of pAkt and pmTOR in BM-MSCs. Meanwhile, hypoxic injury decreased the phosphorylation of mTOR substrates, such as p70S6K and S6, which shows no difference with previous study [8]. Overall, these results suggested that hypoxia downregulated the activation of Akt and mTOR signal pathways.

Inositol polyphosphates are a diverse group of signal molecules, produced through the sequential phosphorylations with inositol polyphosphate kinases (IPKs) from IP3 [42]. IP3, regulating intracellular calcium release, sequentially phosphorylated to generate IP6 and 5-diphospho-inositolpentakisphosphate (IP7). IP6 produces IP7 catalyzed by inositol hexakiphosphate kinase (IP6Ks) [16, 18]. Although the physiological functions of IPs remain poorly clear, studies confirmed that IP7 regulated many physiological functions, including apoptosis [43, 44]. In addition, previous study revealed that increased IP7 signaling induces the emergence of autophagosomes, indicating that IP7 promoted autophagy [15]. Our data also indicated that hypoxic injury increased production of IP7, autophagy, and apoptosis. Furthermore, we found that TNP, a selective inhibition of IP6Ks, reduced the information of IP7 and decreased autophagy and apoptosis. Taken together, all data suggested that hypoxia upregulated IP7, which activates autophagy and apoptosis of BM-MSCs.

IP7 seems to inhibit Akt signaling and regulate apoptosis. Chakraborty also confirmed that IP7 is a physiological inhibitor of Akt signaling pathway [25]. Thus, our results showed that IP7 mediated the effects on BM-MSCs with hypoxia by inhibiting Akt/mTOR signaling, which was in accordance with previous studies [8].

Although present study has some clinical significance, this also includes a lot of limitations. The cellular H/SD model was considered to be useful for excluding the influence of neural and humoral factors in vivo; the model was limited as an artificial experimental model that cannot fully simulate the ischemic and inflammatory environment in vivo. In addition, the physiological function of IP7 has not been fully clear. Therefore, future studies are essential to determine the exact mechanism in order to understand the hypoxic process of BM-MSCs.

## Conclusions

In a word, the current study demonstrated that hypoxic injury increased autophagy and apoptosis by IP7, which inhibit Akt/mTOR signaling pathway in BM-MSCs, which may provide new ideas for the treatment of MI.

## Abbreviations

Akt: Protein kinase B
BM-MSCs: Bone marrow mesenchymal stem cells
DAPI: 4,6-Diamidino-2-phenylindole
FBS: Fetal bovine serum
H/SD: Hypoxia and serum deprivation
IP6: Inositol hexakisphosphate
IP6KI: Inositol hexakisphosphate kinases
IP7: Inositol pyrophosphates
MI: Myocardial infarction
mTOR: Mammalian target of rapamycin
p70S6K: p70 ribosomal S6 subunit kinase
RFP-GFP-LC3: Red fluorescent protein-microtubule-Green fluorescent protein-microtubule associated protein lightchain3
TNP: N6-(p-nitrobenzyl)purine
TUNEL: Terminal deoxynucleotidy transferase-mediated dUTP nick-end labeling
